# Examples of sex/gender sensitivity in epidemiological research: results of an evaluation of original articles published in JECH 2006–2014

**DOI:** 10.1186/s12961-017-0174-z

**Published:** 2017-02-15

**Authors:** Ingeborg Jahn, Claudia Börnhorst, Frauke Günther, Tilman Brand

**Affiliations:** 1 Department Prevention and Evaluation, Leibniz Institute for Prevention Research and Epidemiology – BIPS, Achterstr. 30, 28359 Bremen, Germany; 2 Department Biometry and Data Management, Leibniz Institute for Prevention Research and Epidemiology – BIPS, Achterstr. 30, 28359 Bremen, Germany

**Keywords:** Epidemiological methods, Sex/gender-based analysis, Good practice example

## Abstract

**Background:**

During the last decades, sex and gender biases have been identified in various areas of biomedical and public health research, leading to compromised validity of research findings. As a response, methodological requirements were developed but these are rarely translated into research practice. The aim of this study is to provide good practice examples of sex/gender sensitive health research.

**Methods:**

We conducted a systematic search of research articles published in JECH between 2006 and 2014. An instrument was constructed to evaluate sex/gender sensitivity in four stages of the research process (background, study design, statistical analysis, discussion).

**Results:**

In total, 37 articles covering diverse topics were included. Thereof, 22 were evaluated as good practice example in at least one stage; two articles achieved highest ratings across all stages. Good examples of the background referred to available knowledge on sex/gender differences and sex/gender informed theoretical frameworks. Related to the study design, good examples calculated sample sizes to be able to detect sex/gender differences, selected sex/gender sensitive outcome/exposure indicators, or chose different cut-off values for male and female participants. Good examples of statistical analyses used interaction terms with sex/gender or different shapes of the estimated relationship for men and women. Examples of good discussions interpreted their findings related to social and biological explanatory models or questioned the statistical methods used to detect sex/gender differences.

**Conclusions:**

The identified good practice examples may inspire researchers to critically reflect on the relevance of sex/gender issues of their studies and help them to translate methodological recommendations of sex/gender sensitivity into research practice.

**Electronic supplementary material:**

The online version of this article (doi:10.1186/s12961-017-0174-z) contains supplementary material, which is available to authorized users.

## Background

During the last three decades, sex and gender biases have been identified in biomedical and public health research [1–3]. Stephenson and McKee wrote, in 1993, that in epidemiology “[*g*]*ender is nearly always treated as a potential confounding variable, the effects of which, if there are any, must be controlled for statistically and then ignored. Gender differences in health per se are seldom given a second glance*” [4]. Although some progress has been made, this statement still holds true for large parts of epidemiological research [5–7].

Sex and gender bias refers to an inadequate representation of men and women in study samples, in research teams, or responsible research positions, as recipients of research funding or as authors of publications [8, 9]. In a broader sense, a comprehensive analysis of biological and social factors associated with being a man or a woman is postulated (sex- and gender-based analysis [10]) to explain or prevent sex- and gender-related health inequalities. Sex and gender bias impinges on the quality of epidemiologic research as it compromises the validity of research findings, leading to inaccurate conclusions about how women (or men) respond to a disease and inadequate treatment decisions [9]. From a feminist social science perspective, Eichler proposed four types of gender bias [11, 12], namely gender insensitivity (ignoring gender aspects), over-generalisation (generalisation of research results to a group that has not been studied), double standard (e.g. by drawing on gender stereotypes for explaining gender differences), and androcentrism (male as the norm). Beery and Zucker [1] used the term ‘sex bias’ referring to both inappropriate representation of men and women and neglected differences in biological mechanisms between males and females. Overall, evidence from different research fields suggests that sex and gender bias leads to gaps in the knowledge base and inequalities in the provision of healthcare – in many cases to the disadvantage of women [13, 14].

A central element of sex/gender sensitive research is the distinction between sex and gender. Although the terms ‘sex’ and ‘gender’ denote different aspects of being male and female, they are often used interchangeably in the literature [15–19]. Sex refers to biological characteristics such as chromosomes, hormones or reproductive organs commonly associated with being male or female. Gender refers to sociocultural attributes and related expositions commonly associated with being a man or a woman, such as personality traits, socially constructed roles, access to the labour market, or relative power [17]. However, scholars have warned that the sex–gender dichotomy may mask the complexity, interaction and entanglement between the two concepts [20–22]. To avoid simple dualisms between men and women, other scholars have drawn the attention to the within-group variations at the intersections of sex/gender with other social categories, such as socioeconomic position, race/ethnicity, or age [23, 24]. Krieger [25] pointed out that clear conceptual models are needed for considering sex and gender, simultaneously, and their interrelations (‘biologic expressions of gender’, ‘gendered expression of biology’), “*Yet, we do not live as a ‘gendered’ person one day and a ‘sexed’ organism the next; we are both, simultaneously, and for any given health outcome, it is an empirical question, not a philosophical principle, as to whether diverse permutations of gender and sex matter – or are irrelevant*.” For this purpose, a clear understanding of sex and gender as distinct but entangled concepts and consistent usage of terms are basic requirements of sex/gender sensitive epidemiology [22, 23, 25, 26].

In accordance with other authors [18, 22, 26–30], we prefer to use the shortened version ‘sex/gender’ in this article instead of ‘sex and gender’ or ‘sex and/or gender’. By using the slashed version we want to indicate that sex and gender are distinct concepts, while the slash stands for the potential interrelations between biological and sociocultural aspects of being a man, a woman, or a sex/gender diverse person.

To reduce sex/gender bias, concepts such as ‘engendering epidemiology’ [31] or ‘gendered epidemiology’ [24] were proposed and methodological requirements for sex/gender sensitive epidemiological research were developed [13, 22–24, 32–37]. However, these concepts are rarely translated into research practice [23, 24, 32–35, 37]. Yet, paying attention to sex/gender-related aspects has also been implemented as a requirement in research funding schemes as of the European Union [38] and in science publishing [39]. Thus, applying sex/gender sensitive methods is of increasing relevance for health researchers and epidemiologists [34, 36, 37]. In order to increase sex/gender sensitivity of epidemiological research, it might be useful to present good practice examples of sex/gender sensitive health research. We defined good practice examples as sections of published academic articles that have successfully met methodological requirements of sex/gender sensitivity or that have explicitly described sex/gender-related research practices. Good practice examples can inspire researchers aiming to increase sex/gender sensitivity of their studies and help them to translate methodological recommendations of sex/gender sensitivity into research practice [15].

This study was part of the ‘Epi goes Gender’ project in Germany [36] and aimed to identify recent examples of good sex/gender sensitive research practice illustrating how to apply sex/gender sensitive principles in health research. To this end, we evaluated sex/gender-related research articles published in the *Journal of Epidemiology and Community Health* (*JECH*). We selected *JECH* as the basis for our search due to the journal’s scope (social medicine, social epidemiology) and its major contributions to the field of sex/gender sensitive epidemiology, including the supplement entitled ‘Engendering Epidemiology’ in 2007 and further important publications in this field [13, 40–42].

## Methods

### Search strategy

To identify good practice examples of sex/gender sensitive research, we used the following search strategy. Inclusion criteria were (1) published in *JECH* between 2006 and 2014 to focus on recent development, (2) original research, and (3) title contains ‘sex’ or ‘gender’. The rationale behind this search criterion was to increase the probability to discover articles explicitly dealing with sex/gender aspects [31]. Sex and gender were both included as search terms because we aimed to find examples for biological and sociocultural aspects – and their interrelations. Our aim was not to separate articles dealing with sex from those dealing with gender because, ideally, good practice examples consider both concepts. Articles were excluded if the term ‘sex’ did not denote being male or female or sex-related biological factors but was used in another meaning, e.g. sex(uality), sex work, sex ratio (of newborns). Furthermore, methodological, theoretical and political papers were excluded. The reason for this targeted scope of our search was that we did not aim to provide a comprehensive review of sex/gender sensitivity in epidemiological research, but to illustrate sex/gender sensitivity from purposefully selected examples. Overall, 73 papers were identified that included the terms ‘sex’ or ‘gender’ in their titles. Thereof, 37 articles met all inclusion criteria and were included in the evaluation (Table 1).

**Table 1:** Number of identified, excluded and included papers

*Journal of Epidemiology and Community Health*	N
Sex and/or gender [Title], 2006/01/01 – 2014/12/31 [Date - Publication]	73
Excluded papers	
• the title word ‘sex’ meant something other than the differentiation between males and females, women and men (e.g. sex ratio of offspring, sex work)	24
• methodological, theoretical, political papers	12
Included papers	37

### Assessment instrument

**Fig. 1:**
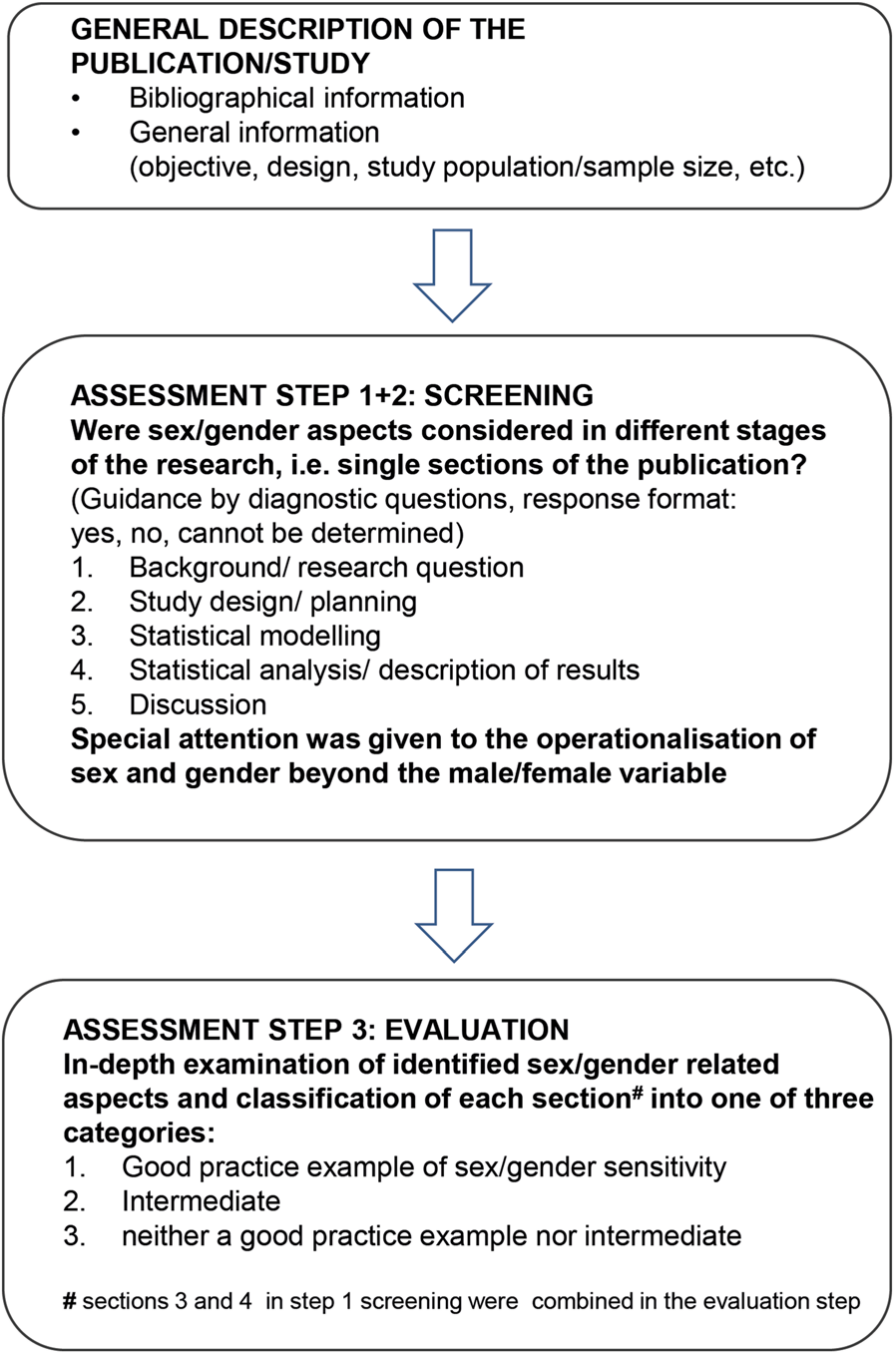
Structure and content of the assessment instrument

We constructed an instrument to evaluate the sex/gender sensitivity of the research presented in the selected articles (Fig. 1 and Additional file 1). Three basic assumptions of sex/gender sensitivity guided the development of the instrument, namely (1) sex/gender-related aspects should be reflected in all stages of the research process [33–35, 43], (2) a sound theoretical conceptualisation of sex/gender is necessary, including the complexity, interaction and entanglement between the two concepts as well as within group variations at the intersections of sex/gender with other social categories such as socioeconomic position, ethnicity or age [23–25, 42, 44–46], and (3) appropriate analytic strategies are to be used [47, 48].

Although a clear distinction between sex and gender is important, our focus was more on how biological and sociocultural factors were included and less on the correct and consistent use of the terms sex and gender.

The evaluation of the full texts comprised three steps. In a first step, each main stage of the research process, represented by the sections of the article (background, study design, statistical analysis, discussion), was screened for addressing any sex/gender-related aspects. In a second step, specific attention was drawn to the operationalisation of sex/gender beyond the binary male/female category. The third step included an in-depth examination of the identified sex/gender-related aspects and a classification of each section into one of three categories: ‘good practice example’, ‘intermediate’, ‘neither a good practice example nor intermediate’. Article sections were classified as ‘good practice example’ if it became apparent that sex/gender concepts guided the related stage of the research process. The category ‘intermediate’ was chosen if only some sex/gender aspects were addressed. The article sections were classified as ‘neither a good practice example nor intermediate’ if sex/gender differences or similarities were not addressed or addressed without any further justification, for example, the sole presentation of sex/gender stratified results. To guide the evaluation, the concept of diagnostic questions [43] was applied. The diagnostic questions were mainly derived based on Eichler et al. [43] and Hammarström [49]. Drafts of the assessment instrument were revised after consultation of experts qualified in epidemiology, public health and gender research. The final version was pretested in a multidisciplinary group of associates of the project ‘Epi goes Gender’ in 2012. The assessment instrument was a generic one and thus not specific to any field of epidemiologic research or study design. It should be highlighted that it was not our aim to assess the quality of the articles in terms of their contributions to their specific fields of research.

### Evaluation

To achieve intersubjective validity, articles were independently evaluated by two evaluators. The first evaluation was completed by CB or FG, both biometricians and trained on the job in principles of sex/gender sensitive health research. The second evaluation was completed by IJ, a social scientist working in the field of sex/gender sensitive health research as a senior researcher and principal investigator of the ‘Epi goes Gender’ project. The instrument was filled in based on the full text without considering any additional information such as design papers or supplementary material. In case of discordant evaluations, a consensus was achieved by communicative validation. In addition to the evaluation, contents of the good practice examples were extracted to illustrate how sex/gender sensitivity was achieved.

## Results

We will first give a rough summary of the results and will then describe good practice examples identified for the different sections of the articles. The topics of the included articles were quite diverse as can be retrieved from article titles [50–86].

In 32 out of 37 articles, sex/gender-related aspects were explicitly named among the research aims, which can be grouped as follows: (1) investigation of the relationships between gender (inequalities) and health [50, 58, 62, 67, 69, 75]; (2) analysis of sex and/or gender differences [51, 52, 54–56, 59, 66, 68, 70, 71, 78, 83, 86]; (3) effect modification analysis by sex/gender [53, 61, 76]; (4) investigation of sex/gender-based origins of health outcomes [57, 60, 63, 73, 77, 80, 81, 84, 85]; and (5) exploration of gender bias [82].

### Results of the screening (steps 1 and 2)

Sex/gender aspects were addressed at least to some extent in 32 out of 37 background sections and in 31 out of 37 discussion sections. In the study design section, 19 out of 37 articles addressed sex/gender aspects. In all included articles, statistical analyses were adjusted for or stratified by sex/gender.

In the second step, we analysed whether sex and gender were operationalised beyond a binary male/female category, i.e. whether more than a single indicator for being male or female was used such as societal indicators of gender relations (e.g. gender equality index) or biological markers (e.g. hormone status). This was the case in 11 articles [51, 53, 58, 63, 66–71, 83] for gender and in 5 articles [54, 55, 60, 62, 81] for both sex and gender. In 21 articles [50, 52, 56, 57, 59, 61, 64, 65, 72–80, 82, 84–86] sex/gender was operationalised as male/female only.

### Results of the comprehensive assessment (step 3)

Twenty-two out of 37 articles were rated as good practice example in at least one section and 2 articles achieved highest ratings across all four sections (Table 2).

**Table 2:** Assessment of sex/gender sensitivity in each section of the selected articles – Synopsis of the results

Reference	Background	Study design	Statistical analysis	Discussion
1. Bambra et al. [50]	++	+	○	++
2. Berntsson et al. [51]	++	++	+	++
3. Boone-Heinonen & Gordon-Larsen [52]	+	○	+	+
4. Borrell et al. [53]	○	○	+	++
5. Escribà-Agüir et al. [54]	++	++	+	+
6. Escribà-Agüir & Artazcoz [55]	++	++	+	+
7. Gissler et al. [56]	+	○	○	+
8. Haukenes et al. [57]	○	○	○	+
9. Harryson et al. [58]	+	++	+	+
10. Hernanadez & Pressler [59]	+	○	+	+
11. Heys et al. [60]	++	++	+	○
12. Hollander et al. [61]	++	○	+	+
13. Ikeda et al. [62]	++	+	+	++
14. Kavanagh et al. [63]	++	+	++	++
15. King et al. [64]	○	○	○	+
16. Kolarcik et al. [65]	○	+	+	+
17. Kovess-Masfety et al. [66]	++	+	+	++
18. Mansdotter et al. [67]	++	++	++	++
19. Matheson et al. [68]	++	+	++	++
20. Matheson et al. [69]	++	+	++	+
21. Matheson et al. [70]	+	+	+	+
22. Matheson et al. [71]	+	+	○	+
23. McCormack et al. [72]	+	○	+	○
24. Milner et al. [73]	+	○	+	+
25. Mindell et al. [74]	○	○	○	○
26. Nante et al. [75]	++	+	+	++
27. Niclasen et al. [76]	+	○	○	○
28. Pitel et al. [77]	+	○	+	+
29. Ratner et al. [78]	+	+	+	+
30. Regidor et al. [79]	○	○	+	○
31. Rigby & Dorling [80]	○	○	○	++
32. Rosenstock et al. [81]	++	++	++	++
33. Ruiz-Cantero et al. [82]	++	++	+	+
34. Staehelin et al. [83]	++	○	○	++
35. Strand et al. [84]	+	○	+	++
36. Värnik et al. [85]	++	○	○	+
37. Vigna-Taglianti et al. [86]	+	○	○	++

Legend: ++ = good practice examples of sex/gender sensitivity, + = intermediate category (sex/gender aspects addressed to some extent); **○** = neither a good practice example of sex/gender sensitivity nor intermediate category

In 14 out of 37 articles at least one section was classified as intermediate category. Only one article was rated as ‘neither a good practice example nor intermediate’ in each section. The proportion of good practice examples was highest for the background (17/37) and discussion (14/37) sections. In the study design and the statistical analysis sections, 6 out of 37 and 5 out of 37 articles were identified as good practice examples, respectively.

#### Background section

All 17 good practice examples for this section referred to available knowledge on sex/gender differences. Some authors referred to gender-informed theoretical frameworks or explanatory models such as the multiple role model, role expansion theory [51, 67], homemaker hypotheses [61], gendered socialisation in cross-cultural comparisons [62], societal gender equality [66], or biological and social explanatory hypotheses for neonatal mortality [81]. The investigation of Heys et al. [60] was explicitly driven by biological differences leading to sex specific effects on lipids and fat patterning. Some authors critically reflected on the used research methods, e.g. averaging effect estimates across subgroups and thereby masking potential effect modifications [63, 68].

#### Study design section

Good practice examples for this section included the following elements: (1) selection of outcome variables that reflect sex/gender differences, (2) calculation of sample sizes enabling the detection of sex/gender differences, (3) selection of sex/gender sensitive exposure indicators, or (4) choosing specific cut-off values for exposure/outcome classifications for male and female participants. For example, Rosenstock et al. [81] applied sex- and gender-based operationalisations of the outcome variable and of influencing factors referring to the entanglement concept [22]. Heys et al. [60] selected the outcome variables based on biological mechanisms concerning the role of sex steroids and growth hormones during puberty. Ruiz-Cantero et al. [82] conducted a pilot study to obtain prevalences for their sample size calculation to attain sufficient statistical power for the detection of differences between men and women in the main study. Harryson et al. [58] as well as Mansdotter et al. [67] selected their exposure variable (work in the domestic and public sphere) based on concepts of gender equality and constructed a gender equality index. Escribà-Agüir et al. [54] and Escribà-Agüir and Artazcoz [55] used different cut off-values for men and women of the Edinburgh Postnatal Depression Scale as prior research had indicated that the threshold for men is two scale points lower compared to women.

#### Statistical analysis section

Good practice examples for this section explicitly accounted for sex/gender aspects in the statistical analyses. This included, for example, the derivation of specific measures accounting for sex/gender differences in exposure or outcome variables [67]. Models were not only stratified by or adjusted for the sex/gender variable (male/female), but further included, for example, interactions with biological or social factors to disentangle sex/gender differences [63, 81]. Conducting a sex/gender sensitive analysis could also mean that different models were fitted to unravel the effects of biological, social and environmental factors [68, 81]. In one example, the shape of the estimated relationship between alcohol consumption and neighbourhood deprivation was allowed to be non-linear and to vary between men and women [69].

#### Discussion section

Good practice examples for the discussion section provided interpretations of their findings by drawing on possible sex/gender-related explanatory approaches or theoretical models [51, 66–68, 81, 83]. Likewise, methodological issues were addressed [63, 67, 75]. For example, in their analysis of neighbourhood deprivation effects on health, Kavanagh et al. [63] critically reflected on the statistical methods used to detect differences between men and women and compared stratified analyses with analyses using statistical interactions. While stratified analyses separately assess the effects among women and men, statistical interactions indicate differences in the effects between women and men. This example highlights the clarity about the question whether effects of exposures in separated groups or differences of effect sizes across groups are to be determined that is needed for the definition of research questions and for subsequent statistical modelling.

## Discussion

This study provides a collection of good practice examples of sex/gender sensitivity based on an evaluation of sex/gender-related health research in a selected scientific journal. Good practice examples were identified in four article sections/stages of the research process and in studies with different aims and designs. The results include examples for many methodological issues that have been discussed in the context of sex/gender sensitive research [23, 24, 32–34, 36, 37]. More good practice examples were found in the background and discussion sections than in the study design and statistical analysis sections. It is interesting to note that only one example (in two articles) was identified where different cut off-values for men and women were used [54, 55]; one other example conducted a pilot study to obtain prevalences in males and females for a subsequent sample size calculation [82].

To the authors’ knowledge this is the first study aiming to identify examples of good practice of sex/gender sensitive research in epidemiology and thus providing practical ideas of sex/gender sensitivity for researchers based on existing science. The presentation of good practice examples demonstrates how sex/gender sensitivity can be accomplished. This may encourage researchers and reviewers to carefully reflect on their research and reviewing practice and could enhance the inclusion of sex/gender aspects in future research.

Our approach can be located in the context of the ‘Gendered Innovations Framework’ [46]. In accordance with this framework, our aim was to illustrate how concepts such as sex and gender can be integrated into the different stages of the research process [46]. The ‘Gendered Innovations’ website [87] offers information for sex/gender sensitive methodological principles in biomedical and public health research and has recently been adopted by the EU Horizon 2020 funding scheme [88]. Currently, ‘Gendered Innovations’ provides seven case studies from medicine and public health illustrating challenges of sex/gender sensitive research, such as the identification of relevant sex/gender factors and their interactions in colorectal cancer or a critical sex/gender analysis of a dietary assessment using a food frequency questionnaire.

New guidelines to advance sex/gender reporting by the European Association of Science Editors are currently under way [39]. In this context ‘sex and gender questions’ were recently published [89], which can be used as a checklist when planning a study. There is a substantial overlap between these questions and the diagnostic questions we used in our assessment instrument. Furthermore, the questions emphasise that both the consideration or not of sex/gender aspects should be justified.

The research topics of the included articles were quite diverse. The evaluators’ expertise covered several research fields but not all topics of the included articles. As a consequence our approach was a generic one, focusing on sex/gender-related aspects but not evaluating the quality of the articles with respect to the state of the art in the specific research field. However, this generic approach enables us to demonstrate that high-quality sex/gender sensitive research can be conducted in a broad area of health research.

Based on the diagnostic questions of our assessment instrument and our findings in the evaluated articles, we developed a checklist of practical steps towards sex/gender sensitivity across the stages of the research process (Table 3). In the right column of Table 3 the steps are illustrated by examples from one research article that achieved high ratings across all four sections. The practical steps do not cover all aspects that have been discussed in the field of sex/gender sensitive research, but can also be used in combination with other concepts or guidelines [22, 39, 90–93].

**Table 3:** Illustrated checklist of practical steps of sex/gender sensitivity in the stages of the research process

Stage of research process - Practical steps	Example from [81]: Sex Differences in Neonatal Mortality in Sarlahi, Nepal: The Role of Biology and Environment
1. Background/Research question	
1.1. Review of existing sex/gender-based knowledge - Are there differences/similarities between and within sex/gender groups? - What are the biological and social causes? - Are there different results across time, space or cultures?	- Differences were found in the literature, e.g. between boys and girls in neonatal mortality in high-income countries (boys are at greater risk) and South Asia (sometimes girls experience more neonatal mortality), and in the early (days 1–7) and late (days 8–28) neonatal period - Biological explanations favouring survival of girls (height/weight, maturity of the lungs, sex steroid influences of the immune system), more relevant in high-income countries - Sociocultural explanations for girls’ risk of neonatal mortality in South Asia: gender preference, differential care-seeking behaviours, birth order and family composition, perceptions of illnesses
1.2. Evaluation of the knowledge base. What is the sex/gender-related gap?	“*Unanswered questions remain regarding the impact that biological (immutable factors specific to the newborn or his/her mother) and environmental factors (mutable external factors) have on sex specific trends in neonatal mortality*” [81]
1.3. Formulation of sex/gender-related study aim and research question to address the knowledge gap	“… *biological and environmental factors that might explain sex differences in neonatal mortality*…” [81]
2. Study design	
2.1 Definition of sex/gender-related biological and social factors based on a theoretical model	- Biological factors typically indicating a higher risk for neonatal mortality in males: birth outcomes such as weight, gestational age, respiratory depression, malformations - Social/environmental factors which may indicate a gender preference: peri- and postnatal care such as feeding practices, hygiene and skin care practices, warming practices and care-seeking behaviours
2.2 Selection of sex/gender sensitive outcome and exposure measures	- Sex/gender-based justification of the outcome measure early/late neonatal mortality
2.3 Sample size calculation is justified with respect to sex/gender-related study aims, e.g. to detect differences between or within sex/gender groups	- Secondary analysis of a population-based randomised trial, 23,662 newborns were included in the analysis
3. Statistical analysis	
3.1 Analytic strategy, statistical modelling is justified with respect to the sex/gender-related aims of the study	- Stratified analysis by sex/gender and ethnicity, explorative examination of sociodemographic, newborn and maternal characteristics; model building strategy reflected the four conditions: biological vs. social/environmental factors, early vs. late neonatal period
3.2 The analysis is conducted stratified by sex/gender (if appropriate) but avoids overemphasis of sex/gender	- Differentiation by ethnic groups (Pahadi and Madeshi)
3.3 Sex/gender stratified presentation of sample characteristics	- Sociodemographic characteristics are reported to not be meaningfully different between boys and girls
3.4 Sex/gender differences and similarities are reported	- Biological factor, care practices and crude mortality rates were presented by sex/gender and differed significantly - Multivariate models analysing biological and social/environmental factors in the early and the late neonatal period showed no influence of care related factors - Further exploration showed social factors in one ethnic group to be related with excess mortality in the late neonatal period
4. Discussion	
4.1 Findings are discussed in the context of existing literature; unexpected results, strength and weaknesses of the study with regard to sex/gender aspects are interpreted	- Main results are discussed with regard to: • Expectations concerning early vs. late neonatal period • Seasonal influences on food availability for pregnant women • Newborn care services favoured boys, providing evidence of gender preference • Differences within the group of girls depending on ethnic group (Pahadi, Madeshi) and prior sex composition of siblings - Missing values on birth weight are discussed as a limitation, but did not affect sex/gender-related factors
4.2 Implications for research and practice of the main sex/gender-related findings are discussed	- Important issues are highlighted: (1) neonatal analysis must be stratified by early and late period, (2) biology has a greater impact on early, environmental factors on late neonatal mortality, (3) the explanation model ‘gender preferences’ is oversimplified as it applies only to a certain group

Several measures were taken to establish intersubjective validity such as diagnostic questions to provide an orientation, double ratings and consensus building strategies. Nevertheless, the professional background of the evaluators may have had some influences on the assessment, for example, assigning the operationalisation of the variables to the statistical modelling part or to the study design. Searching for sex or gender in titles only may be viewed as a limited approach. However, comparing this strategy with an extended search including the abstracts of the articles revealed that the strategy was very successful in identifying studies that explicitly aim to investigate sex/gender-related aspects [94]. A further limitation could be that we did not examine supplementary materials or related papers of the included studies. These materials may have contained more information on aspects of the study design such as the validity of the measures and sample size calculations. However, our expectation was that the key elements of the study design would be published in the original article.

## Conclusion

This study provides good practice examples of sex/gender sensitivity in epidemiological research. The examples include different lines of research, such as questions of gender research in epidemiology, e.g. influences of individual or societal gender relations on health, as well as questions of the relevance of sex/gender aspects for the definition of exposures, outcomes and respective pathways (sex/gender-based analysis). While gender research presupposes a specific epistemological perspective and research interest which may not be shared by many epidemiologists, sex/gender-based analysis is relevant to the quality of epidemiological research as it contributes to our understanding of causal pathways. Determining the relevance and well-founded inclusion or exclusion of sex/gender aspects and the careful epidemiological analysis is an important requirement for a more equitable provision of healthcare and preferably gender transformative [95] preventative services for women and men.

Measures to motivate and enable researchers to increase sex/gender sensitivity should focus on aspects of the study design and the statistical analysis, e.g. sex/gender sensitive selection of determinants and outcomes, operationalisation of sex/gender aspects, and power calculation.

Next steps in the analysis of sex/gender sensitivity in epidemiological research would include the coverage of a broader range of epidemiological journals extracting both positive examples as well as examples of sex/gender bias. This should also include an assessment of the consistency and conceptual clarity of the terms sex and gender [96].

### What is already known?

- Epidemiological research is often of limited significance in terms of sex/gender sensitivity.
- There is a growing body of knowledge concerning the analysis and avoidance of sex/gender bias.

### What this paper adds?

This paper serves as a first step to use positive examples in order to develop and disseminate sex/gender sensitive research and provides indications on how to practically implement sex/gender aspects.

## Additional file

Additional file 1: Assessment instrument: Identification of good practice examples of sex/gender sensitive health research. (PDF 48 kb)

## Abbreviations

JECH: Journal of Epidemiology and Community Health
