# Supplementary material for: Examples of sex/gender sensitivity in epidemiological research: results of an evaluation of original articles published in JECH 2006–2014
Source: Health Res Policy Syst. 2017 Feb 15;15:11. doi: 10.1186/s12961-017-0174-z (PMC5312447; doi:10.1186/s12961-017-0174-z)
Supplement: Additional file 1: — Assessment instrument: Identification of good practice examples of sex/gender sensitive health research. (PDF 48 kb) [file 12961_2017_174_MOESM1_ESM.pdf]

"How are sex/gender aspects considered in current research in epidemiology?"

Identification of good practice examples of sex/gender sensitive health research

Project "Epi goes Gender": Evaluation of current publications – evaluation sheet (19.06.2012)

The aim of evaluating current publications in epidemiological journals is to identify positive examples of the consideration of sex/gender aspects in epidemiological literature. It is not proposed to value the publication as a whole but the evaluation is based on the reading of the whole article. The criteria of the evaluation focus on the consideration of the sex/gender-category in different stages of the research process reported in the publication. Based on the works of Eichler, Fuchs and Maschewsky-Schneider [1] and Doyal [2], [3], reference will be made to the conceptual clarity regarding the operationalisation of sex/gender, the methods of analysis and the interpretation/ conclusion.

The term "sex" refers to the biological characteristics commonly associated with being a men or a woman. Biological characteristics refer to the physiological and anatomic differences or rather similarities of men and women. Differences exist regarding hormones, primary and secondary sex characteristics as well as sex chromosomes.
For example: Men generally have more massive bones than women; due to differences in metabolism and particular enzymes, the decomposition of alcohol is slower in women than in men, and women recover faster than men from anaesthesia.

The term "gender" refers to the different social, cultural and psychological dimensions of sex/gender as e.g. masculinity and femininity. This also includes social roles, rights and duties, living circumstances and life styles, the self-identification as male or female or characteristics that are attributed to men and women (sex stereotypes). Cultural and social constructions of being a woman, man, girl and boy are not static but changeable.
For example: In many countries women earn significantly less money than men for similar work; in Vietnam more men than women smoke, as smoking is traditionally considered inappropriate for women; in most parts of the world, women do more housework than men.

Sources: [4-6]

For each article the following data will be assessed:

1. Bibliographical information

2. General information

3. Description and evaluation of the consideration of sex and/or gender aspects

Advices for completion:

The questionnaire can be completed electronically as a word document or on paper.

_____	= Free text of any length (only if completed electronically, otherwise please use an extra sheet)
☐	= Check-box
[ ]	= Choice box (only if completed electronically, otherwise complete on paper; for number of authors, publication year and journal)

1. Bibliographical Information

1.1 Title of the article: (please write the complete title)

1.2 Number of authors:

____

1.3 Name and sex of the main authors:

	Last name	First name	Sex
**First author**			m☐ f☐
**Second author**			m☐ f☐
The second author will be recorded if the first and second author equally contributed to the publication.			
**Last author**			m☐ f☐

1.4 Affiliation of the corresponding author:

1.5 Journal:

______

1.6 Year of publication:

_____

2. General information

2.1 Objective: (Please describe in plain text)

2.2 Outcome: (Please describe in plain text)

2.3 Factors of influence considered: (multiple answers possible)

☐ social situation/ socio-economic factors
☐ occupational factors
☐ environmental factors
☐ smoking/ passive smoking
☐ alcohol consumption
☐ physical activity
☐ nutrition
☐ migration
☐ health care utilisation
☐ biological factors (genes, hormones, anatomic differences)
☐ other factors, which ones:

2.4 Basis for the classification of men and women: (multiple answers possible)

☐ self-report
☐ register
☐ passport/ residents' registration office
☐ other, which ones (e.g. appearance)
☐ not specified

2.5 Design/ study type: (multiple answers possible)

☐ meta-analysis

☐ randomised controlled trial

☐ intervention study

☐ cohort study

☐ case-control study

☐ cross-sectional study

☐ analysis of secondary data

☐ qualitative research

☐ design not identifiable

2.6 Does the article refer to any theoretical and/or conceptual framework?

☐ Yes, the authors formulate/propose/describe/built up a conceptual framework describing/explaining the associations between analysed factors and outcomes (e.g. bio-psycho-social framework, bio-medical framework, social scientific framework with or without explicit and implicit consideration of sex/gender aspects).

Please describe the framework briefly:

☐ No

☐ Unsure/ do not know

2.7 Study population: (Please enter what is given in the article)

Description of the study population: (multiple answers possible)

☐ children aged less than 6 years
☐ children aged 6-13 years
☐ adolescents aged 14-17 years
☐ adults aged 18-39 years
☐ adults aged 40-64 years
☐ adults aged 65 years and older
☐ others (e.g. workers, insured persons)
☐ children/ adolescents without information on age
☐ adults without information on age
☐ no information

Sample size:

**Male**	**Female**	**Total**
(n)	(n)	(n)
%	%	100%

2.8 Main results of the study: (Please write in plain text)

3. Assessment of the consideration of sex and gender aspects

The evaluation is carried out in three steps:

Step one (3.1): screening question to generally assess whether "sex and/or gender aspects" were considered "anyhow" separately for five stages of the research process (Background/research question, Study design/planning, statistical modelling, statistical analyses/description of the results, Discussion/conclusion).

Step two (3.2): operationalisation of sex/gender-factors.

Step three (3.3): evaluation of the consideration of sex and gender aspects regarding four different stages of the research process.

3.1 Consideration of sex and/or gender aspects during the different stages of the research process, respectively in the sections of the publication

This question is supposed to get a general impression on whether there is anything to find about "sex and/or gender". The evaluation is carried out separately for five different stages, or rather sections. We included some diagnostic questions (derived mainly from [1, 7]) for each of the five stages/ sections, which are supposed to guide you through the evaluation. The answer YES includes the explicit as well as the implicit consideration of sex and/or gender aspects. Here, explicit consideration means that sex/gender aspects are included with a clear explanation while implicit consideration means that the study includes sex/gender aspects with no specific explanation regarding sex/gender.

1. Background/ research question:

Are sex/gender aspects considered in this section?

☐ yes ☐ no ☐ cannot be determined

Reasons for the decision:

Diagnostic questions:

Is the objective relevant for men and women? Is the relevance plausibly explained (e.g. in relation to previous knowledge)?

Are the considered aspects relevant for the target population? Are they equally important for men and women? Are important sex/gender related aspects missing?

Are the main variables explained related to sex/gender? Is there e.g. any description of potential differences between men and women?

Are the theoretical framework, study design and study methods constructed in such a way that sex and/or gender related aspects can be discovered?

2. Study design/ planning

Are sex/gender aspects considered in this section?

☐ yes ☐ no ☐ cannot be determined

Reasons for the decision:

Diagnostic questions:

Were sex and/or gender considered in the power calculation?

Are sex and/or gender specific aspects considered during the survey (e.g. in-/ exclusion criteria)?

Are response rates reported for men and women separately? Are sex and/or gender related aspects considered during the survey (e.g. house wives vs. workers, survey at the supermarket)?

Are the used survey instruments identical for men and women? If not, is an explanation given?

Are the instruments tested for validity and reliability for both, men and women? Is the procedure adequate?

3. Statistical Modelling

Are sex/gender aspects considered in this section?

☐ yes ☐ no ☐ cannot be determined

Reasons for the decision:

Diagnostic questions:

Is the applied modelling adequate? (Are similar models used in similar studies, e.g. reference to literature on the model?)

How are sex and/or gender aspects considered in the statistical modelling? (q.v. questions of diagnostic of background/ objective)

4. Statistical analyses/ description of results

Are sex/gender aspects considered in this section?

☐ yes ☐ no ☐ cannot be determined

Reasons for the decision:

Diagnostic questions:

Are sex and/or gender considered in the statistical analyses?

If the analyses are not conducted stratified for men and women, has been checked beforehand, whether this is plausible?

Are the study population and the results separately described for sex and/or gender? Is the procedure adequate?

5. Discussion

Are sex/gender aspects considered in this section?

☐ yes ☐ no ☐ cannot be determined

Reasons for the decision:

Diagnostic questions:

Are sex and/or gender aspects considered in the interpretation or the limitations?

Are the results interpreted with reference to theoretical frameworks as e.g. gender theories?

Are the conclusions drawn compatible with the results? Are all relevant sex and/or gender aspects discussed?

Have inadequate generalisations been made? (E.g. if a study analyses only males and generalizes for females - without justification or if a study analyses both males and females in middle age and generalizes for all adults.)

3.2 Please evaluate the conceptual clarity regarding the operationalisation of sex and gender

An operationalisation of sex can only be affirmed if biological aspects are specified as comprehensively as possible; an operationalisation of gender can only be affirmed if socio-cultural aspects are specified as comprehensively as possible. It is not sufficient if "just something" is mentioned about sex/gender. [3]

The simultaneous consideration of sex and gender refers to the entanglement of biological and social aspects of being a man or woman [5]. For a 'yes', the entanglement between sex and gender should be explicitly considered.

Were sex and/or gender aspects operationalised beyond the simple classification male/female (see 2.4)?

1. Was sex assessed/ included explicitly (e.g. genes, hormones)?[1]

☐ no

☐ yes, which aspects:

---

2. Was gender assessed/ included explicitly (e.g. gender-related life situations, behaviour)?

☐ no

☐ yes, which aspects:

---

3. If sex and gender is included explicitly (1. and 2. Yes), are there interactions between sex and gender considered (e.g. interaction of coffee or alcohol consumption patterns and biological aspects)?

☐ no

☐ yes, which aspects:

Reasons for the decision: (if necessary differentiated for 1.-3.)

---

[1] These questions refer only to the fact whether a factor was considered or not. An assessment on the relevance regarding the research question is not intended.

3.3 Overall evaluation:

How is the overall evaluation of the sections of the publication? Does it provide examples of (good) sex/gender sensitive practice and/or at least "starting points" (intermediate category) for sex/gender sensitive studies in the future?
Please evaluate each of the four parts mentioned below!

	**"Example": well founded consideration of sex and/or gender aspects**	**Intermediate category[2]**	**"No example":**
**Background/ research question**	☐	☐	☐
**Study design/ planning**	☐	☐	☐
**Statistical modelling/ analysis**	☐	☐	☐[3]
**Discussion**	☐	☐	☐

3.4 Sureness of the overall evaluation:

☐ **(rather) sure**

☐ **(rather) unsure**

Notes regarding the overall evaluation you would like to add: (optional)

Please note here what you consider as important reasoning for your evaluation, e.g.:

- *Explanation for the evaluation*
- *Missing information from your perspective (e.g. modelling, methodology, presentation of results, discussion)*
- *Explanation for the "unsure" mark*
- *General characteristics: e.g. regarding language or structure of the article*
- *You can also refer to the reasoning you gave for the evaluation of questions 3.1 + 3.2.*

---

[2] The Intermediate category was introduced for cases, which consider sex/gender aspects, but without explaining/justifying these in the context of sex and gender.

[3] Also applies if the analysis was conducted only stratified for "surveyed sex" (e.g. self reported sex, sex in passport/ questionnaire/ databank). This is affirmed if statistical modelling/ analysis neither was evaluated as example of (good) sex/gender sensitive practice nor as intermediate category.

Literature:

1 Eichler M, Fuchs J, Maschewsky-Schneider U. Richtlinien zur Vermeidung von Gender Bias in der Gesundheitsforschung. *Zeitschrift für Gesundheitswissenschaften= Journal of public health* 2000;8:293-310.
2 Doyal L. Sex and gender: the challenges for epidemiologists. *Int J Health Serv* 2003;33:569-79.
3 Doyal L. Sex und Gender: Fünf Herausforderungen für Epidemiologinnen und Epidemiologen. *Gesundheitswesen* 2004;66:153-7.
4 Jahn I. Die Berücksichtigung der Geschlechterperspektive. *Bundesgesundheitsblatt Gesundheitsforsch Gesundheitsschutz* 2005;48:287-95.
5 Krieger N. Genders, sexes, and health: what are the connections--and why does it matter? *Int J Epidemiol* 2003;32:652-7.
6 Kuhlmann E, Kolip P. Gender und Public Health. Weinheim: Juventa 2005.
7 Hammarström A. A tool for developing gender research in medicine: examples from the medical literature on work life. *Gend Med* 2007;4 Suppl B:S123-32.
